# Jejunogastric Intussusception after Pylorus Resecting Pancreaticoduodenectomy: A Rare Case Report and Review of the Literature

**DOI:** 10.70352/scrj.cr.25-0621

**Published:** 2026-01-28

**Authors:** Yuma Tamaki, Hideki Motobayashi, Atsushi Shimizu, Akihiro Takeuchi, Kyohei Matsumoto, Shinya Hayami, Atsushi Miyamoto, Kensuke Nakamura, Manabu Kawai

**Affiliations:** Second Department of Surgery, Wakayama Medical University, Wakayama, Wakayama, Japan

**Keywords:** jejunogastric intussusception, pancreaticoduodenectomy, Child procedure complication, emergency operation

## Abstract

**INTRODUCTION:**

Jejunogastric intussusception is a rare complication that can occur following gastrectomy or gastric bypass surgery. The occurrence of intestinal intussusception after pancreaticoduodenectomy (Child reconstruction) has been reported in very few cases in the literature.

**CASE PRESENTATION:**

Here, we present the case of a 75-year-old patient who developed jejunogastric intussusception following pancreaticoduodenectomy performed for intraductal papillary mucinous neoplasm of the pancreatic head. The diagnosis was confirmed by contrast-enhanced abdominal CT and upper gastrointestinal endoscopy. During the operation, the efferent limb was intussuscepted into the gastric lumen. Manual Hutchinson’s maneuver of the intussusception was successfully performed without incision of the stomach or jejunum. Intestinal blood flow was assessed during surgery using indocyanine green fluorescence imaging and adequate blood flow was confirmed. The surgery was completed without intestinal resection or incision of the stomach or jejunum. The patient’s postoperative course was uneventful, and he was discharged in stable condition on POD 11. He showed no recurrence at follow-up 1 year after the surgery.

**CONCLUSIONS:**

Although jejunogastric intussusception is an uncommon complication following pancreaticoduodenectomy, it can lead to life-threatening outcomes. Delayed diagnosis can necessitate bowel resection. Prompt diagnosis and emergent surgical intervention are essential for effective treatment, highlighting the importance of a rapid clinical response from diagnosis to treatment regarding jejunogastric intussusception.

## Abbreviations

ICG
: indocyanine green
JGI
: jejunogastric intussusception

## INTRODUCTION

JGI is a rare complication of gastrectomy or gastric bypass surgery.^1)^ The reported incidence rate is extremely low at 0.1%.^2)^ JGI after pancreaticoduodenectomy is an extremely rare but potentially life-threating complication. Emergency surgery based on prompt diagnosis is therefore critical to prevent bowel ischemia and necrosis. However, physical findings might be sometimes subtle and nonspecific, although clinical presentation typically includes acute abdominal pain, hematemesis, nausea, vomiting, and signs of gastric outlet obstruction. As another problem, if bowel ischemia is detected during surgery, surgeons sometimes wonder whether they can preserve the intestine. Intestinal resection after reconstruction of pancreaticoduodenectomy is generally a highly complex procedure, so determining the necessity for intestinal resection is critically important. However, no reports in the literature feature large series or offer guidelines.

Here, we present a rare case of JGI that developed after pylorus-resecting pancreaticoduodenectomy. By using intraoperative ICG fluorescent imaging, emergency surgery successfully preserved the ischemic intestine, resulting in a favorable clinical outcome. Additionally, we reviewed case reports in the literature regarding JGI after pancreaticoduodenectomy to evaluate surgical management as the standard of care for JGI after pancreaticoduodenectomy.

## CASE PRESENTATION

**Fig. 1:**
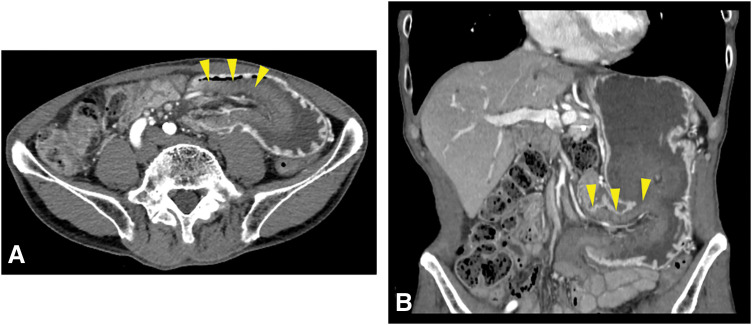
Contrast-enhanced abdominal CT in both an axial image (**A**) and a coronal image (**B**) revealed jejunal loop intussusception at the gastrojejunal anastomosis (yellow arrowheads), which had evidence of edematous wall with slightly poor enhancement of the intussuscepted loop.

**Fig. 2:**
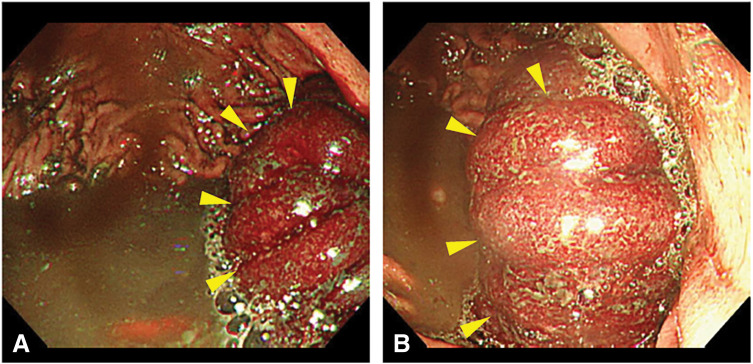
Emergency upper gastrointestinal endoscopy confirmed invagination of the small intestine (yellow arrowheads) into the stomach in both a far view (**A**) and a near view (**B**).

**Fig. 3:**
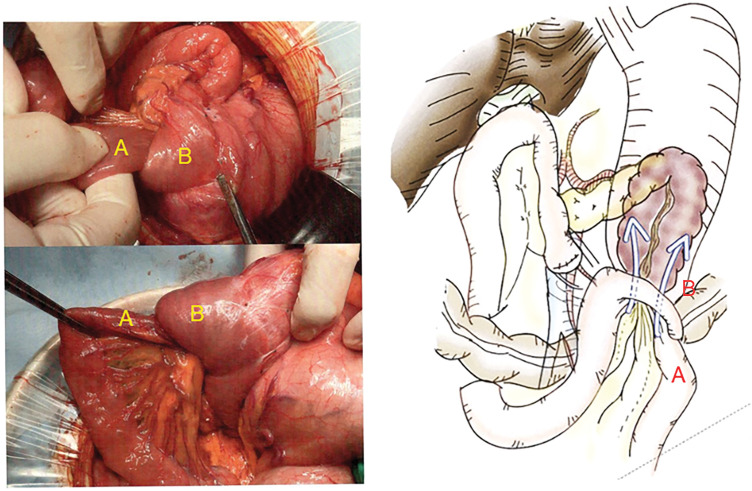
Intraoperative findings revealed jejunogastric intussusception. Invagination of the efferent limb (A) into the stomach (B) through the jejunogastric anastomosis. The size of jejunogastric anastomosis was approximately 5 cm.

**Fig. 4:**
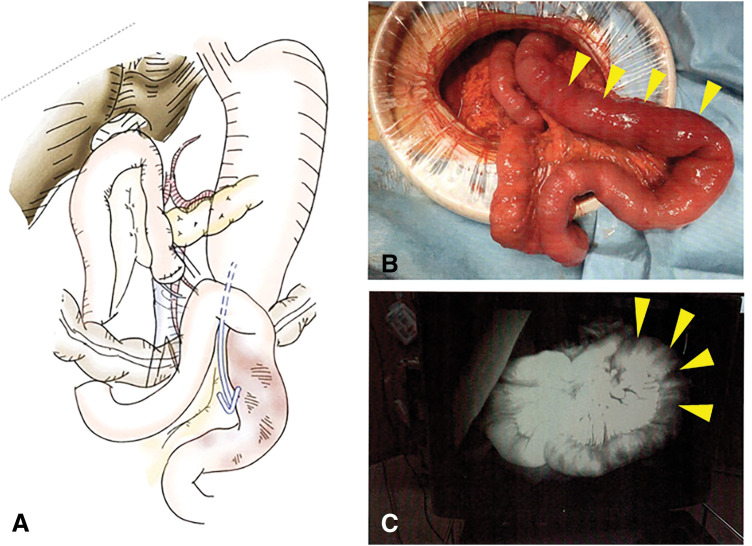
(**A**) The scheme showed that manual Hutchinson’s maneuver of the intussusception was successfully performed without incision of the stomach or jejunum. (**B**) The part of the efferent limb with slightly poor color in the operative field indicated by the yellow arrowheads. (**C**) Intestine blood flow in the efferent limb (yellow arrowheads) which was macroscopically of slightly poor color was confirmed by using indocyanine green fluorescence imaging.

A 75-year-old man presented to the emergency department with epigastric pain and black-colored vomit. He had undergone pylorus-resecting pancreaticoduodenectomy with Child reconstruction without Braun’s anastomosis 4 years previously for intraductal papillary mucinous neoplasm of the pancreatic head. Gastrojejunostomy was performed via the antecolic route. He was followed up every 3 months for the first 3 years by blood examination and CT, and every 6 months thereafter at our department. During follow-up at our department, he presented to our institution as an emergency patient. This episode was the first event for him. His vital signs were stable, and axial body temperature was 36.5°C. On examination, spontaneous and tender pain was noted in the epigastric region. Laboratory tests showed no obvious inflammatory response such as white blood cell count or C-reactive protein, and there were no other remarkable findings except for a slight elevation in pancreatic enzymes. Contrast-enhanced abdominal CT revealed intussusception of the jejunum into the gastric lumen at the site of gastrojejunostomy, with edematous changes of the involved bowel loop (**Fig. 1**). Emergent upper gastrointestinal endoscopy confirmed invagination of the small intestine into the stomach, with mucosal discoloration due to congestion (**Fig. 2**). Endoscopic reduction using insufflation was attempted but unsuccessful, and emergency surgery was subsequently performed. During the operation, the efferent limb was intussuscepted into the gastric lumen (**Fig. 3**). The size of jejunogastric anastomosis was approximately 5 cm. Manual Hutchinson’s maneuver of the intussusception was successfully performed without incision of the stomach or jejunum (**Fig. 4A**). No apparent necrosis or ischemic changes of the small intestine were observed intraoperatively. Although transient ischemia might just have occurred due to the intussusception, the color of the efferent limb which was intussuscepted in the area indicated by the arrowheads in **Fig. 4B** appeared slightly poor. Therefore, ICG fluorescence imaging was performed to objectively evaluate intestinal blood flow. Afterward, adequate intestinal blood flow was confirmed by ICG in **Fig. 4C**. The part of the efferent limb with slightly poor color in the operative field indicated by the arrowheads in **Fig. 4B** corresponded to the ICG fluorescence image indicated by the arrowheads in **Fig. 4C**. As the protocol for the dosage and timing of the ICG administration, ICG (7.5 mg) was intravenously administered, and fluorescence was observed approximately 20 seconds after injection to evaluate the blood flow of intestine. The surgery was completed without intestinal resection or incision of the stomach or jejunum. The patient’s postoperative course was uneventful, and he was discharged in stable condition on POD 11. He showed no recurrence at follow-up 1 year after the surgery.

## DISCUSSION

We reported the successful preservation of ischemic intestine by emergency surgery in a patient with JGI 4 years after pylorus-resecting pancreaticoduodenectomy. It was facilitated by intraoperative ICG fluorescent imaging. JGI was first reported in a case following distal gastrectomy,^3)^ but reports of JGI after pancreaticoduodenectomy are exceedingly rare; since the first report in 2007,^4)^ only 13 cases have been reported to date, including this one.

We reviewed these 13 cases,^4–14)^ including our case, and their details are summarized in **Table 1**. The time to intussusception from pancreaticoduodenectomy varied greatly: from as little as 6 days to as much as 5 years. Among them, 8 patients (61.5%) underwent reconstruction by the Whipple method, 3 (23.1%) by Child procedure, and 2 case reports (15.4%) did not specify the reconstruction method. The 11 cases (84.6%) involved intussusception of the efferent limb into the gastric lumen, while 1 (7.7%) involved the afferent limb. In all 13 reported cases, the patients required emergency surgery. Intestinal resection was necessary in 8 cases (61.5%), whereas the intestine was preserved in the remaining 5 cases (38.5%). Among 8 cases with intestinal resection, 4 cases underwent Roux-en-Y reconstruction following intestinal resection, however, in the remained 4 cases, no detail for the surgical methods of reconstruction after the intestinal resection was described. Our case is thought to be notable for the successful reduction of the patient’s intestinal intussusception by combining the Hutchinson maneuver with gentle traction of the intestine without incision of the stomach wall or gastrotomy. No case studies reported the recurrent intussusception following manual reduction without intestinal resection. However, long-term follow-up studies after manual reduction without intestinal resection have not been conducted. Caution should be required regarding the recurrence of intussusception after manual reduction without bowel resection, although no recurrence of intussusception has been observed over 1 year of follow-up in our case. Although there are the 4 cases (30.8%) in which organ preservation was possible, this is the first report to show that intestinal preservation is possible by using ICG fluorescent imaging to evaluate intestinal blood flow during surgery.

**Table 1:** Summary of 13 cases with jejunogastric intussusception after pancreaticoduodenectomy

Author	Year	Age/sex	Diagnosis	Surgical procedure	Reconstruction	Time after PD	Brynitz classification	Surgical procedure (method of reconstruction)
Gigena et al.^4)^	2007	40/M	Pancreas divisum and chronic pancreatitis	PD and Puestow procedure	Whipple	4 years	Type 2a	Resection and reconstruction (Roux-en-Y)
Patel et al.^5)^	2019	37/M	P-NET	PD	Whipple	9 months	Type 1	Resection and reconstruction (Roux-en-Y)
Lyu and Xu^6)^	2020	68/M	Ampullary cancer	PD	Whipple	5 years	Type 2a	Reduction (without incision of stomach anterior wall)
Zhou et al.^7)^	2020	67/M	Pancreatic cancer	RAPD	Child	6 days	Type 2a	Resection and reconstruction (N/M)
Moore et al.^8)^	2020	68/M	Pancreatic cancer	PD	Whipple	3 years	Type 2a	Resection and reconstruction (Roux-en-Y)
Yogo et al.^9)^	2022	81/M	Distal bile duct cancer	PD	Child	3 years	Type 2a	Reduction (with incision for stomach anterior wall)
Khuong et al.^10)^	2023	54/M	Distal bile duct cancer	RAPD	Whipple	14 months	Type 2a	Resection and reconstruction (N/M)
Khuong et al.^10)^	2023	59/M	Pancreatic ductal adenocarcinoma	PD	Whipple	5 years	Type 2a	Resection and reconstruction (Roux-en-Y)
Zhang et al.^11)^	2023	66/W	N/M	PD	N/M	N/M	Type 2a	Reduction (without incision for stomach anterior wall)
Marable et al.^12)^	2024	70/W	Pancreatic cancer	PD	Whipple	2 years	Type 2a	Reduction (without incision for stomach anterior wall) and resection of small bowel and reconstruction (N/M)
Martinez-Esteban et al.^13)^	2024	60/M	Distal bile duct cancer	PPPD	Whipple	2 years	Type 2a	Resection and reconstruction (N/M)
Sotomayor et al.^14)^	2025	41/M	Non-functional neuroendocrine tumor	LPD	N/M	3 years	N/M	Reduction (without incision for stomach anterior wall)
Our case	2025	75/M	IPMN	PRPD	Child	4 years	Type 2a	Reduction (without incision for stomach anterior wall)

IPMN, intraductal papillary mucinous neoplasm; LPD, laparoscopic pancreaticoduodenectomy; N/M, not mentioned; PD, pancreaticoduodenectomy; P-NET, pancreatic neuroendocrine tumor; PPPD, pylorus-preserving pancreaticoduodenectomy; PRPD, pylorus-resecting pancreaticoduodenectomy; RAPD, robotic-assisted pancreaticoduodenectomy

JGI following Billroth II gastrectomy has been classified into 5 types based on the intussuscepted limb: Type 1 involves the afferent limb invaginating into the remnant stomach, Type 2a involves the efferent limb invaginating into the remnant stomach, Type 2b describes the efferent limb intussuscepting into itself, Type 3 involves both afferent and efferent limbs invaginating into the stomach, and Type 4 involves intussusception at the Braun’s anastomosis.^15)^ The gastrojejunostomy performed in pancreaticoduodenectomy resembled Billroth II reconstruction, so the present case corresponds to Type 2a in Brynitz’s classification, which is the most common JGI after Billroth II gastrectomy.

The etiology of JGI remains unclear. Large anastomotic sites and abnormal intestinal motility have been reported as factors that contribute to the occurrence of JGI following pancreaticoduodenectomy, total gastrectomy, or partial gastrectomy.^16–18)^ Other proposed causes include elongated afferent or efferent limbs and increased intra-abdominal pressure.^16–18)^ In the present case, the diameter of the gastrojejunostomy anastomosis was 5 cm. As previous case reports regarding jejunogastric intussusception did not report the anastomotic diameter, it is not possible to evaluate whether the 5 cm length in this case was longer or not compared with those in previous cases. The anastomotic size of gastrojejunostomy may need to be equal to the caliber of the duodenum to prevent jejunogastric intussusception or dumping syndrome. Furthermore, although we take care to ensure that the afferent and efferent legs are not too long and to avoid tension on the anastomosis during gastrojejunostomy, the afferent and efferent loop lengths are not measured during gastrojejunostomy in pancreaticoduodenectomy at our institution. Therefore, whether the cause of intussusception is the size of the anastomosis or the length of the afferent and efferent legs remains a matter of speculation and cannot be fully discussed. Small intestinal peristalsis is known to be driven by interstitial cells of Cajal located in the duodenum.^19)^ Pancreaticoduodenectomy involves resection of the duodenum, so abnormal small bowel motility may be induced, which could potentially contribute to the development of JGI. Notably, most reported cases of JGI after pancreaticoduodenectomy involve intussusception of the efferent limb rather than the afferent limb, which may be explained by the absence of interstitial cells of Cajal leading to retrograde peristalsis.

We performed contrast-enhanced abdominal CT for diagnosis in the present case, and this was also done in all 12 previously reported cases of post-pancreaticoduodenectomy JGI. Contrast-enhanced CT remains an essential diagnostic tool for prompt identification of this rare complication. Among the 12 previously reported cases of JGI after pancreaticoduodenectomy, bowel preservation was achieved in only 4 cases (33.3%). Two of these 4 cases involved fixation of the reduced intestine to the afferent limb, while the 2 cases did not specify whether fixation to adjacent organs was performed. In our case, no fixation to other organs was performed after reduction, and no recurrence of intussusception has been observed over 1 year of follow-up. Regarding bowel preservation, the decision on whether to preserve the intestine can sometimes be extremely difficult during operation. At our institution, we use ICG for visualization and quantification of anastomotic perfusion in colorectal surgery.^20,21)^ Based on this assessment, we propose its utilization as a decision-making criterion for constructing diverting stoma in cases of concern regarding poor blood flow at the anastomotic site. This is the first report of utilization of ICG for small bowel perfusion assessment in JGI following pancreatoduodenectomy. ICG fluorescence visualization and quantification are recommended as a useful tool for assessing the viability and potential for preservation of intestine, and also in emergency surgery involving JGI.

## CONCLUSIONS

JGI is an uncommon complication following pancreaticoduodenectomy, but it can lead to life-threatening outcomes. Delayed diagnosis can necessitate bowel resection. Prompt diagnosis and emergent surgical intervention are essential for effective treatment, highlighting the importance of a rapid clinical response from diagnosis of JGI to treatment.

## DECLARATIONS

### Availability of data and materials

The data that support the findings of this study are not openly available due to reasons of sensitivity but are available from the corresponding author upon reasonable request.
